# Electronic Nose Differentiation between *Quercus robur* Acorns Infected by Pathogenic Oomycetes *Phytophthora plurivora* and *Pythium intermedium*

**DOI:** 10.3390/molecules26175272

**Published:** 2021-08-30

**Authors:** Piotr Borowik, Leszek Adamowicz, Rafał Tarakowski, Przemysław Wacławik, Tomasz Oszako, Sławomir Ślusarski, Miłosz Tkaczyk, Marcin Stocki

**Affiliations:** 1 Faculty of Physics, Warsaw University of Technology, ul. Koszykowa 75, 00-662 Warszawa, Poland; pborow@poczta.onet.pl (P.B.); Rafal.Tarakowski@pw.edu.pl (R.T.); Przemyslaw.Waclawik@pw.edu.pl (P.W.); 2 Forest Protection Department, Forest Research Institute, ul. Braci Leśnej 3, 05-090 Sękocin Stary, Poland; T.Oszako@ibles.waw.pl (T.O.); S.Slusarski@ibles.waw.pl (S.Ś.); M.Tkaczyk@ibles.waw.pl (M.T.); 3 Institute of Forest Sciences, Faculty of Civil Engineering and Environmental Sciences, Bialystok University of Technology, ul. Wiejska 45E, 15-351 Białystok, Poland; m.stocki@pb.edu.pl

**Keywords:** odor classification, VOC, volatile organic compounds, fungi and biosecurity

## Abstract

Identification of the presence of pathogenic oomycetes in infected plant material proved possible using an electronic nose, giving hope for a tool to assist nurseries and quarantine services. Previously, species of *Phytophthora plurivora* and *Pythium* intermedium have been successfully distinguished in germinated acorns of English oak *Quercus robur* L. Chemical compound analyses performed by HS-SPME/GC-MS (Headspace Solid-Phase Microextraction/Gas Chromatography–Mass Spectrometry) revealed the presence of volatile antifungal molecules produced by oak seedlings belonging to terpenes and alkanes. Compounds characteristic only of *Phytophthora plurivora* or *Pythium intermedium* were also found. Methylcarveol occurred when germinated acorns were infected with *Pythium*, while neophytadiene (isomer 2 and 3) occurred only when infected with *Phytophthora*. Moreover, isopentanol was found in acorns infected with *Phytophthora*, while in control, isopentyl vinyl ether was not observed anywhere else. Among the numerous volatile compounds, isopentanol only occurred in acorns infected with *Phytophthora* and methylcarveol in acorns infected with *Pythium*.

## 1. Introduction

With the increase in international trade of plants and plant source materials, such as seeds, new threat are arising from the accidental introduction of insects or pathogens into new environments. Their spread can be responsible for massive damage to local ecosystems. Issues with the health of forest tree species occur already at a very early stage of their establishment in nurseries. Significant reductions in seedling quantity and associated economic losses are caused by damping-off diseases caused by soil-borne fungi as well as oomycetes such as *Phytophthora* and *Pythium*. Even surviving asymptomatic plants that do not exhibit visible external disease symptoms may still carry pathogenic inoculum such as chlamydospores between root systems in the soil. Diagnostic tests conducted in many European countries ahve revealed that infestation of seedlings is high, in some cases reaching 80 per cent [1]. Since most fungicides are not able to control oomycetes, species identification is crucial for the development of adequate control, mainly due to the emergence of fungicide-tolerant isolates of oomycetes in horticulture and agriculture [2,3,4]. In addition, identifying potential hosts and the particular location of their occurrence in a nursery allows managers to avoid potential infection of plants by appropriate crop rotation. For example, they can grow acorns from oaks in locations where *Phytophthora alni* has been found, as it does not cause them serious harm, and conversely grow alder seeds where *Phytophthora quercina* has been found. Unfortunately, pathogenic oomycetes are often transferred to other environments (e.g., riparian areas) with plating along rivers of asymptomatic seedlings. Therefore, scientists and plant health inspectors need new, efficient tools like electronic noses (e-noses) to act early and efficiently.

The simplest method of detecting oomycetes in soil is to lay out bait plants. The procedure involves infecting pieces of leaves (e.g., oak or beach leaves), allowing the pathogens to grow, and then applying them in selective media such as PARP. (PARP is a specific medium with antibiotics, thanks to which the fungi do not grow because they are sensitive, but *Phytophthora* is resistant to it [5].) It is usually a lengthy process. Such an approach requires several days or weeks to obtain pure cultures that can be used to identify pathogenic oomycetes by classical (microscopic) or molecular (DNA sequencing) methods. One of the complications of baiting is the need to use different host leaves, and that isolation temperatures may be different for different organisms.

It has been reported [6] that trained dogs can detect *Phytophthora* species by sniffing. It would be helpful to follow this idea and use an artificial device for a similar task. This is an ambitious goal and our first experiments, reported in reference [7], were designed and performed in vitro under controlled laboratory conditions. We prepared sample growth on a traditional medium. We succeeded in showing that the e-nose we designed can be helpful to discriminate between two different but closely related oomycetes. According to our experience, such an achievement was not self-evident since distinguishing between *Phytophthora* and *Pythium* samples often leads to false-positive results even in molecular tests, e.g., ELISA (enzyme-linked immunosorbent assay). Furthermore, it is complicated when both microorganisms are present in the same environment, e.g., in soil.

In the second part of the experiment reported in this paper, we prepared samples more similar to natural conditions, as the oomecytes were cultured on germinated acorns. In this case, we expected the volatile odour components associated with the samples to differ from the standard growth medium. In addition to potential odour molecules emitted by pathogen metabolism, odours from oak seedlings and secondary metabolites emitted by the infested plant are also present.

Many analytical techniques can be used for the detection and analysis of odours. Gas chromatography with mass spectrometry is considered the gold standard of classical chemical analytical techniques and is used successfully under laboratory conditions. However, the high cost of expensive equipment and the hiring of skilled personnel means that this method is not widely used in forestry, agriculture or horticulture. Therefore, there is a need for innovative designs of less expensive instruments capable of detecting organism species by detecting volatile organic compounds (VOCs). In addition, these instruments should be suitable for on-site monitoring in a relatively short measurement time. Technological developments have led to the proliferation of e-noses as rapid and non-invasive diagnostic tools. Since the introduction of the e-nose concept [8,9,10], various methods of measurement and data collection have been developed.

A summary of the potential applications, limitations, challenges and proposed improvements of e-noses in focusing on bacterial, fungal and viral infections is described in reviews [11,12,13,14,15]. Much of the previous research on fungal odours recognition [16] has focused on their properties concerning food and flavour. Recently, reports have been published on studies on *Penicillium expansum* spoilage of apples [17], *Aspergillus* species discrimination [18] and analysis of VOCs of different fungal species. Studies of VOCs of *Phytophthora cactorum* species were reported by Wang et al. [19] and Greenshields et al. [20] in a case of infected strawberries. An electronic nose was used to detect fungal infection of wood [21,22]. There are other reports of studies on the detection of fungal infection in different cereals [23,24,25,26,27,28]. Applications of an electronic nose for detection of fungi in tree roots are also reported [29]. Sahgal et al. [30] presented results on the discrimination of dermatophyte species and strains. Lampson et al. reported the development of a wearable electronic nose for pest and plant damage detection [31].

We want to emphasize the motivation of the presented research. Oaks are among the forest-forming species with which much hope is associated in Poland. However, their area is only about 7% of the entire country and it is still increasing, especially the sessile oak *Quercus robur* L. First, foresters are trying to convert pine monocultures and increase the proportion of other tree species in poor habitats such as fresh forests. Second, climate scientists predict that coniferous species will retreat to northern Europe, where the climate is more suitable for them, and deciduous species, including oaks, will take their place. Oaks bear fruit irregularly every few years, so it is challenging to provide enough seedlings each year to meet the needs of 430 forest districts. In nurseries, acorn and seedling diseases affect dieback in the first period, so germinated acorns were used for the experiment. The pathogen selected in this case was the most common species in Polish forest nurseries, *Phytophthora plurivora*. It is morphologically similar to other oomycetes of the genus *Pythium*, some of which, like *Pythium nunn*, are antagonists of pathogenic species such as *Pythium ultimum*. However, damage to plants by *Pythium* is usually less severe and economically viable, whereas species of the genus *Phytophthora* can destroy entire plantations. For this reason, it is essential for the nurseryman to know which organism he is dealing with and to make a rational decision as to whether to protect the plants or rely on the natural resistance processes of the oaks. The electronic nose can be of great help here, as it is a low-labour device that makes sharp distinctions.

## 2. Materials and Methods

### 2.1. Samples Preparation

The isolates of *Phytophthora plurivora* and *Pythium intermedium* used in the colonisation test were obtained from the oomycete culture collection of the Forest Research Institute (IBL). All isolates were taken from the rhizosphire of declining oaks showing distinct disease symptoms. They were all identified morphologically, with molecular confirmation of the morphological findings.

Non-stratified seeds of *Quercus robur* were used for the different pathogenicity tests. Acorns were incubated in sterilised moist sand at 25 °C under the light. One month later, acorns were removed from the soil, washed under running water and surface sterilised with 70 % ethanol. The species used in the assay *Phytophthora plurivora* and *Pythium intermedium* were transferred to V8A media prepared with 800 mL/L distilled water, 200 mL/L V8 juice (Tymbark, Poland), 18 g/L agar-agar (BTL, Poland) and 3 g/L CaCO${}_{3}$. Inoculum of both species was obtained from the growth margins of 3–4 day-old colonies incubated at 22–25 °C in the dark [32]. Agar plugs containing mycelium (approximately 1 cm${}^{2}$ in size) were placed in sterilised, 200 mm glass Petri dishes with sterile filter paper. Tips of *Quercus robur* acorns with a radicle length of 5–7 cm were placed on agar plugs with mycelium in Petri dishes. Seedlings in individual Petri dishes were used for each variant. The control group also consisted of four seedlings and was placed on the sterile agar plugs. Filter paper in the dishes was moistened with 5 mL of sterile distilled water and the dishes were incubated under daylight at approximately 20–22 °C. The dishes were monitored every 8 h until the first seedlings collapsed and the first necroses were observed. When necrosis was observed on all the individual roots, the acorns were transferred from the Petri dish to a jar and left there for the entire duration of the measurements. From this point on, measurements of volatile odorants were made. According to Koch’s postulates, re-isolation of the pathogens from the roots was performed. The applied procedure ensured no undesirable contamination of the sample with other organisms during inoculation. To reisolate *Phytophthora plurivora* and *Pythium intermedium* from the falling tissues, small necrotic parts of the seedlings (0.1–0.2 mm in size) were cut with a sterile razor blade and plated on selective media (V8A-PARPNH) prepared according to Jung et al. [33]. Tissue fragments from the control group were also plated on V8A-PARPNH media.

**Figure 1:**
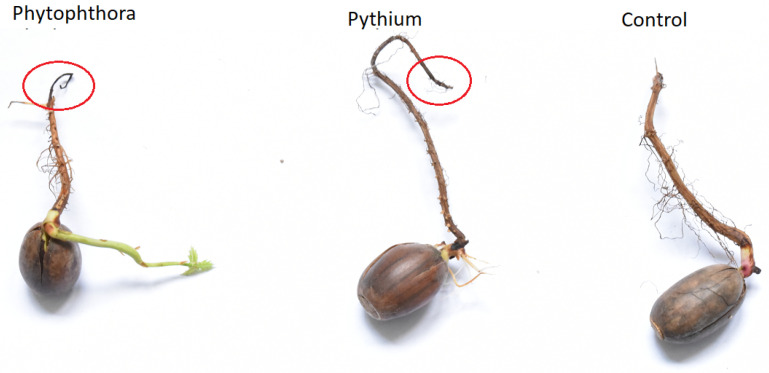
Examples of measured samples of three considered categories: Healthy acorn (Control), acorn infected by *Phytophthora*, acorn infected by *Pythium*. Tissue necrosis can be distinguished from healthy regions in infected samples.

Photography of examples of measured samples, in which necrosis in infected samples is remarkable and visually differentiated from the healthy organisms, is presented in Figure 1. For this photography, we selected only one example of acorn from each category. The main aim was to demonstrate the variability of the samples and the relatively small size of the necrosis.

### 2.2. Headspace Solid-Phase Microextraction and Gas Chromatography-Mass Spectrometry (HS-SPME/GC-MS) Analysis

Volatile metabolites of acorns were analysed by HS-SPME/GC-MS method. In preliminary studies of VOCs emitted from oak acorns (control), the comparison of divinylbenzene/carboxen/polydimethylsiloxane (DVB/CAR/PDMS), CAR/PDMS and PDMS sorption fibres (Supelco, Bellefonte, PA, USA) was performed. The best effectiveness of the extraction–desorption cycle was obtained by DVB/CAR/PDMS fibre. Therefore this type of SPME fibre was used in further research. Analyses were performed according to a previously developed methodology [34,35,36,37], as described below.

Acorns were placed into a 60 mL vial and heated for 60 min at 40 °C. The membrane of the screw cap was pierced with the needle containing the SPME fibre and the fibre was exposed to a headspace gas phase for 30 min at 40 °C. Immediately after exposure, the SMPE fibre was placed in an injection port of the GC-MS instrument for 10 min to desorb the volatiles from the SPME fibre thermally. The injector was operated at a temperature of 250 °C in splitless mode. GC-MS analyses were performed using an Agilent 7890A gas chromatograph with an Agilent 5975C mass spectrometer (Agilent Technologies Inc., Santa Clara, CA, USA). Chromatographic separation was performed using a capillary column HP-5MS (30 m × 0.25 mm × 0.25 μm) at a 1 mL/min helium flow rate in constant flow mode. The initial temperature of the column was 35 °C and was increased to 250 °C at a rate of 5 °C/min. The transfer line temperature was 300 °C. The acquisition parameters of the mass spectrometer were as follows: The source temperature of 230 °C and the quadrupole temperature of 150 °C. The electron impact mass spectra were obtained at ionisation energy of 70 eV. The detection was performed in full scan mode for an of 29–600 atomic mass units.

After chromatographic separation, all peaks from the chromatogram were integrationed and the percentage content of components in the total ion current (% of TIC) was calculated. Both the mass spectrometric data and the calculated retention indices were used to identify the components. Mass spectrometric identification was performed using NIST (2020) and Wiley (2020) mass spectra libraries. The retention indices of the compounds were determined considering the retention times of the C5–C40 n-alkanes. The experimental retention indices (RI${}_{exp.}$) were compared with the literature values of retention indices (RI${}_{lit.}$).

### 2.3. Electronic Nose

#### 2.3.1. Device Construction

**Figure 2:**
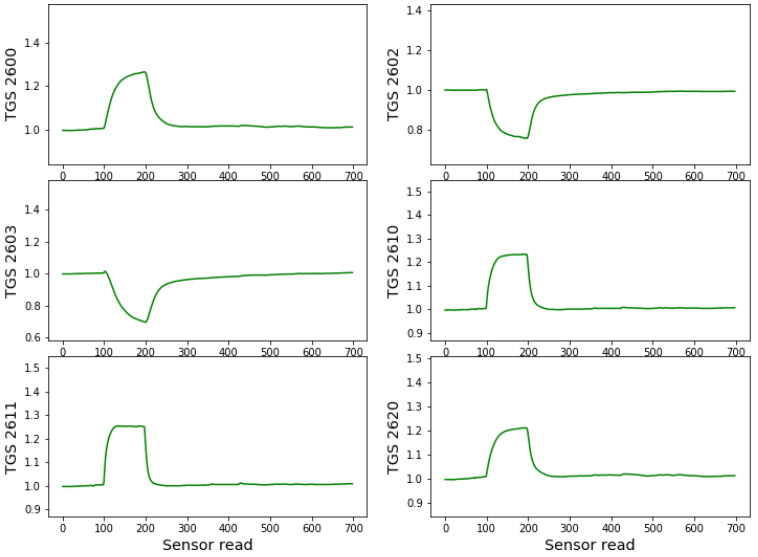
Example of the sensors’ responses (conductance) during one measurement cycle of a sample of healthy acorns. On the x-axis, the number of individual reads of the sensor resistance is used. The sensor data are collected every 1.2 s. The sensors responses are standardised by the baseline value obtained as the average of the first 100 reads of sensors values when sensors were exposed to the clear air conditions.

The low-cost electronic nose device, constructed at Warsaw University of Technology, consists of six metal oxide sensors manufactured by Figaro Inc. (Osaka, Japan): TGS 2600 (air contaminants), TGS 2602 (VOCs, ammonia and H${}_{2}$S), TGS 2603 (amine and sulfur types of odor: Trimethylamine, methyl mercaptan, etc.), TGS 2610 (LP gas), TGS 2611 (methane), TGS 2620 (Organic solvent vapors). The resistance of the sensors depends on the conditions of the gas to which the sensors are exposed. However, the sensors are not strictly selective and they also respond to other gases with different magnitude and different time response characteristics. When measuring with the electronic nose, the response curves are recorded when the conditions suddenly change from clean air to the odorous air under consideration and back to clean air. An example of such time-dependent characteristics of the sensor’s response during one measuring cycle is presented in Figure 2.

**Figure 3:**
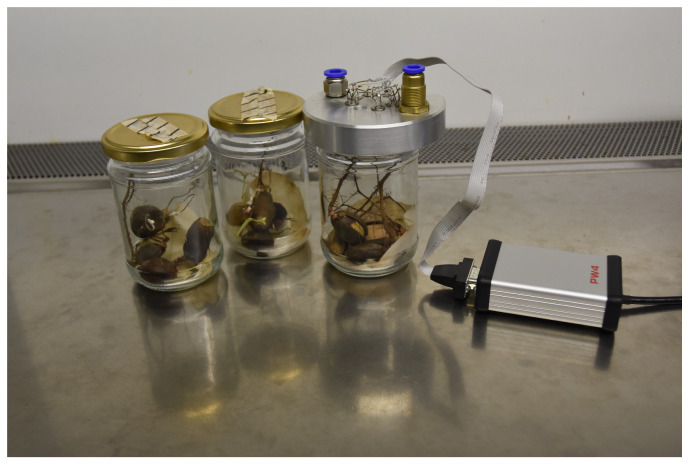
Electronic nose device with measured samples of healthy and infected acorns in jars.

In the electronic nose device, the sensors are mounted in a round metal probe of the diameter of 10 cm, which fits the jars containing the measured samples, as is presented in Figure 3. According to the measurement procedure, the probe can be moved manually and placed in clear air conditions or close to the measured odour source.

The sensors respond to the gas by conductance changes due to oxygen exchange between the material surface and the measured gas. We implemented a voltage divider circuit for each sensor to measure the sensor’s response and the voltage is measured on the serially connected resistor. This measurement circuit topology has been chosen do its simplicity and also as the sensors producer recommendation. However, it should be noticed that other types of measurement circuits can be implemented for the MOS-type electronic noses [38]. The voltage probe took about 20 ms for each sensor. This was repeated 50 times and averaged to reduce the electrical noise at the hardware level. The reading of the voltages from the whole sensors array took about 1.22 s. The MCP3208 12-bit AD converter has been used for the digitisation of the signals. Output data were stored online on the laptop in the text file. All operations of the control of the devices and collection of data were performed on the laptop. Moreover, the power supply to the electronic nose has been provided by the laptop. A detailed description of the device was given in the previous paper [7].

#### 2.3.2. Samples Measurements

The measurement procedure is similar to the one used in our previous research reported in reference [7]. Our experiments performed with the electronic nose device were performed in a laminar flow cabin (Telstar, Bio II Advance) at a temperature of 21 °C with the air supply turned on. That allowed us to keep controlled conditions of constant temperature and humidity during the whole experiment. The MOS gas sensors’ sensitivity depends significantly on these parameters, while we are concerned with finding the differences in sensor response to the odours emitted by the studied samples. Keeping constant humidity allows us to treat sensors’ response to it as a background signal, similar in all cases. Application of the electronic nose for measurements in the field would require a more advanced setup of temperature and humidity control, such as stabilisation of the device temperature and drying measured gas by silica gel. That would significantly increase the complexity and cost of the electronic nose device. Another solution that could be considered is compensation mechanisms on the hardware level, requiring detailed knowledge of sensor response characteristics or on the software level, which would require a much more extensive training dataset than we collected during our experiments.

Figure 3 shows the measurement setup with samples of acorns in jars. For the measurements, we used 4 samples of acorns for each category. The measurements were repeated 31 times for each category, so in total 93 measurements were performed.

In the first phase of each measurement, the sensors were exposed to clean air and the baseline responses of the sensors were recorded. The jar containing an acorn sample was then opened and the electronic nose sensors were held against the closure of the jar for 122 s to record the response curve of the sensors. The sensors were then removed and placed in clean air, which allowed recovery and relaxation to baseline.

In Figure 2 we can observe the response of the example sensors for a measurement cycle of a sample of healthy acorns. As can be seen, during the first 100 readings (122 s), the sensors are in clear air, which is considered baseline. When placed near the sample containing the measured odour source, we can observe an abrupt change in conductance until the 200th reading (122 s). Then again, the electronic nasal probe movement to clear air conditions causes an abrupt change in response characteristics during sensor cleaning and relaxation to baseline conditions. This cleaning/relaxation time was chosen to be 610 s (500 readings of the response values), allowing for complete sensor cleaning and recovery. The sensors are statically exposed (without airflow) to the clean air or measured odour conditions, except for brief moments of manual movements.

**Figure 4:**
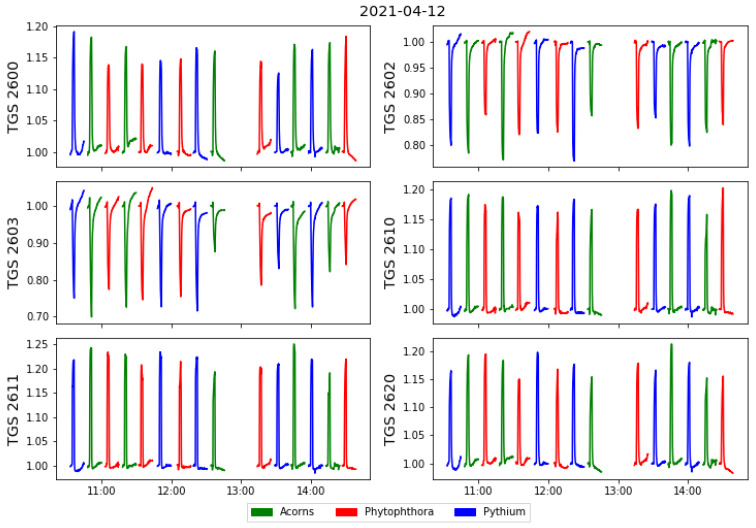
Example of all sensors responses collected during one day of the measurements, versus time of the measurement. The sensors’ responses are standardised by the baseline value obtained as the average of sensors values when exposed to the clear air conditions at the beginning of each measurement cycle.

Several series of measurements were made in one day. The possibility of detecting patterns due to undesirable trends in the measurement setup or external environmental conditions is considered. For that reason order of the measured samples was randomised with a random number generator using an Excel spreadsheet. On six experimental days from 7 to 15 April 2021, 93 measurements were made on different samples. In Figure 4 we show an example of a measurement day, where different colours distinguish the different categories of measured samples.

#### 2.3.3. Data Analysis Techniques

We used two well-established statistical analysis techniques to analyse the data obtained from the gas sensors exposed to the odours emitted by the measured samples. First, we performed the Principal Component Analysis (PCA) to visualise the obtained data, which helps gain intuitive insight into the data distribution and similarity between studied cases. Secondarily, the primary statistical analysis consisted of building machine learning classification models to estimate the applied measurement techniques’ ability to distinguish between the considered cases of samples.

A detailed description of the modelling techniques used is presented in previous works [7,39], however for the reader’s convenience, we would like to provide here an abbreviated description of the performed analysis.

The data analysis presented in this paper was performed using computer codes developed in the Python 3.7 language with the scikit-learn module.

##### Data Visualisation Using Principal Component Analysis

Principal Component Analysis is one of the commonly used statistical methods of factor analysis, allowing to transform the data space to new space represented by factors for which we assume their importance in dataset variability. For example, a dataset of observations defined by *N* variables represents a cloud of points in a *N*-dimensional space. The goal is to find the coordinate system, so the first coordinate represents the direction in which the variance of the data points is maximal, then the second coordinate, which is perpendicular to the first one, capturing the maximum of the remaining variance of the dataset. This can be intuitively interpreted as the rotation of the coordinate system. The coordinate axes transformed in this way are loads of the generated factors (principal components), in which the initial factors explain the most variability of the dataset. PCA is often used to reduce the dimensionality of the dataset by discarding the less critical components. The PCA may be based on either a correlation matrix or a covariance matrix constructed from the input dataset. It can be shown that the principal components are eigenvectors of the correlation/covariance matrix of the dataset observations values. The algorithm of both cases is identical, but the obtained results are different. When the covariance matrix is used, the input variables with the highest variance have the most significant impact on the outcome, which may be desirable if the variables represent comparable quantities. On the other hand, the correlation matrix corresponds to the initial normalisation of the input set to have the same variance.

##### Machine Learning Classification Modelling

In the case of the data of electronic nose measurements, we have sensors response curves for six sensors as presented in Figure 2. However, to the analysis, we do not use the raw data collected by the sensors but more complex modelling features representing various characteristics describing the shape of these curves. It will be described in more detail in the subsequent section. Just as one example, we can notice that we can mention the area under the curve and maximum/minimum values of the response curves. As one can notice, they are represented in different measurement units and their numerical values cannot be directly compared. For such reason, we used normalised values to account for each feature on the same footing.

Our studies used the PCA method only for visualisation purposes, allowing us to gain intuition into the distribution of data in the studied case. As input for this data transformation, we used features describing the shapes of the sensor response curves used for the classification modelling. The most relevant features selected by the classification model were used.

The data collected by the electronic nose can be used to discriminate between the samples under study, which is a classical classification task for which machine learning models are commonly used [40,41]. A well-established methodology was used.

- In the first step, the raw data of the collected sensor responses are transformed into the modelling features describing the shapes of the response curves.
- To estimate the classification models performance, we performed the six-fold cross-validation (CV) procedure. For this task, we applied group splitting, assuring that all data collected during one day of the measurements were put to one of these subsets. Such an approach is commonly observed to correlate measuring conditions due to external measurement conditions such as sensor drift. Moreover, since we are interested in estimating the performance of the classification model for measurements performed in the future, this approach is the most suitable to give reliable estimates. This number of repetitions in the CV loop was determined because our measurements were performed during six days. Thus, the maximal number of splits could ensure the separation of the training and testing subsets by the day.
- The machine learning classification model was applied and the most important features were selected using the recursive forward selection approach [39] when we first select the best performing model based on a single feature and then add to the model subsequent features based on the performance improvement.

It is essential to explain the approach to extracting the modelling features used to build the classification models. As we present in Figure 2, the sensors conductance values, measured as a function of time, as a response to the moving of the sensors from clear air to the measured odour conditions and again to the pure air form characteristic curves. Therefore, it is common to use as modelling features variables describing their shapes [42] instead of just raw sensors data. In our studies, we used several types of such features [7,39]

- The basic characteristics of the response curve include minimum, maximum value, average (which is equivalent to the integral/area under the curve), standard deviation, skewness and kurtosis.
- The exponential moving average (emaα) of the response curve and their maximum/minimum values for several smoothing parameters α are extracted as modelling features [43,44].
- Extreme values of the response curve derivative [45,46] as well as other statistics calculated from the derivative curve such as average, standard deviation, skewness, kurtosis. These features are calculated separately for two parts of the sensor response curve, the adsorption phase, when sensors respond to the measured odour conditions, and the desorption phase when they relax after moving them to the clean air. Moreover, the derivative of the curve is calculated after smoothing by the exponential moving average method.
- Characteristic times, such as the time to reach 10%, 25%, 50%, 90% of the sensor response range and time to reach maximum/minimum of the curve derivative,
- Parameters of sensor response curve fitting by third-order polynomial function, separately for the adsorption and the desorption part [45,46].

We tested different techniques such as Logistic Regression, Support Vector Machine (SVM) and Decision Trees as a machine learning algorithm for classification. As our preliminary tests revealed, the first of the mentioned techniques usually provided the best models from the point of accuracy; however, the difference between them was very close. Our choice was to use the Logistic Regression method for the final calculations.

To evaluate the model performance, we use the most common statistical measures: Accuracy, precision, recall, defined in terms of the entries of the confusion matrix.

(1) $$\begin{array}{ccc} accuracy & = & \frac{tp+tn}{tp+tn+fp+fn^{\prime }} \end{array}$$

(2) $$\begin{array}{ccc} precision & = & \frac{tp}{tp+fp^{\prime }} \end{array}$$

(3) $$\begin{array}{ccc} recall & = & \frac{tp}{tp+fn^{\prime }} \end{array}$$

where the components of the confusion matrix are defined in Table 1.

## 3. Results and Discussion

As explained in the previous section, several types of data analysis were performed. The first part of the research identifies the chemical compounds found in the odour emitted by the studied samples of healthy and infected acorns, performed by the Gas Chromatography-Mass Spectrometry method. In the second part of the described research, the data collected by the electronic nose measurements were used for building classification models. The results of this procedure in terms of the most important variables were visualized after transformation by the PCA method. However, the main results of the classification are the model performance statistics such as accuracy, precision and recall.

### 3.1. VOCs Identified in Emission from Acorns with Use HS-SPME/GC-MS Method

The results of HS -SPME/GC-MS analysis of the three acorn samples were grouped (i) according to chemical compound groups (Table in Appendix A) and (ii) and the VOCs (Table 2) differentiating the studied samples.

Compounds characteristic only of *Pythium intermedium* or *Phytophthora plurivora* were found. Methylcarveol, with relative content of 1.43 %TIC, occurred when acorns were infested with *Pythium*, while isomer 2 and 3 of neophytadiene, with the content of 0.28 and 0.53 %TIC respectively, occurred when acorns were infested with *Phytophthora*. All these chemical compounds belong to the group of terpenes. Isopentanol (0.65 %TIC) occurred when acorns were infested with *Phytophthora* (Table 2). The remaining compounds were characteristic of both control and treatments, implying that they were characteristic of acorns.

The detailed results of the Gas Chromatography-Mass Spectrometry measurements are presented in Appendix A.

### 3.2. Principal Components Analysis of the Electronic Nose Data

**Figure 5:**
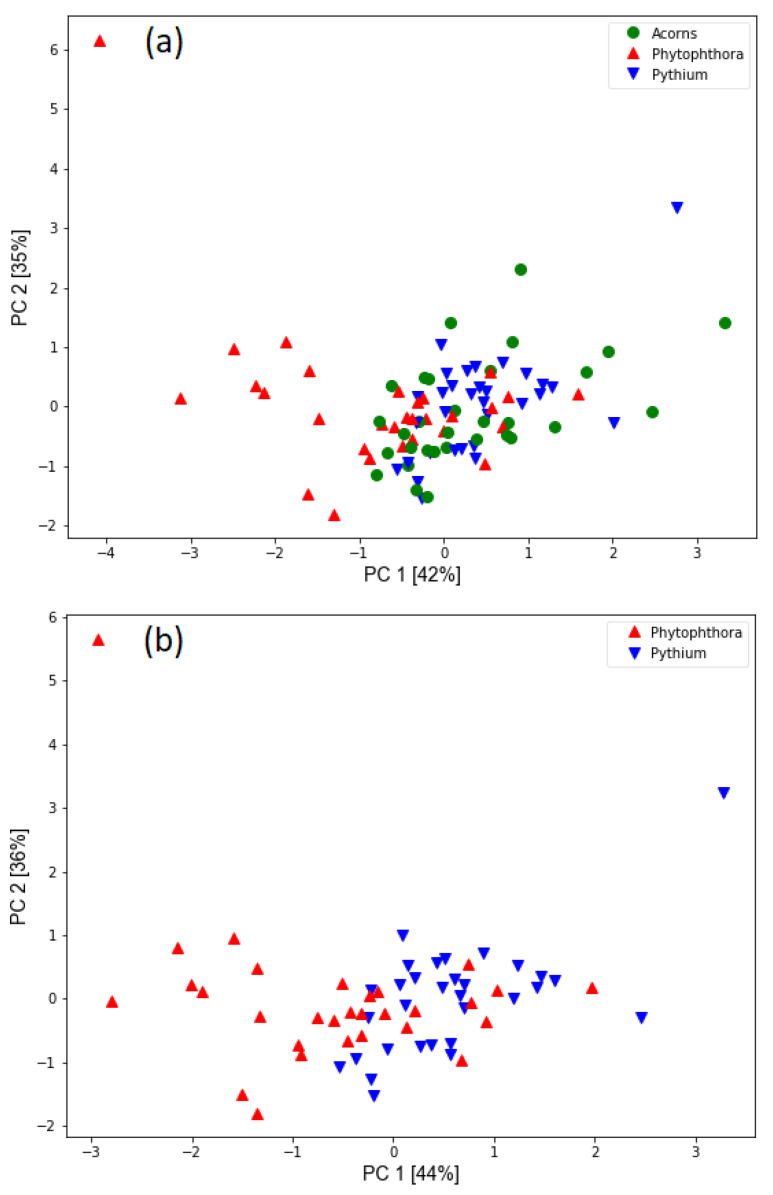
Distribution of measured samples based on principal component analysis transformation of modelling features extracted from sensor response curves. The percentage of variability accounted for by the principal components is indicated in the axis labels. The types of samples measured are represented with different colours and symbols. (**a**) The three categories of measured samples are plotted. (**b**) Only the samples infected with oomycetes are shown to illustrate the difference.

As a first result of the analysis of the data collected by the electronic nose in Figure 5, we present the PCA transformation of the studied dataset when the features selected by the classification algorithm were used as input. The two first principal components are visualized and as we can notice, they contain about 80% of the data variability.

### 3.3. Classification Model Using Electronic Nose Data

As shown in Figure 5, there is considerable overlap in the data from measurements on healthy acorns and acorns infected with *Pythium*. Likely, the seedlings were not infected to the extent that secondary metabolites were produced that could be detected by e-nose. This feature was also confirmed by our further analysis, where we created machine learning models of the models. Probably because of this, we were not able to train a model that could distinguish between healthy acorns and samples infected with *Pythium*. However, this figure also shows the difference between samples infected with two oomycete species of the genus *Pythium* and *Phytophthora*. We trained several classification models and found that it was indeed difficult to distinguish between the tested samples. The performance of the models in terms of accuracy is fragile, giving at most an improvement of an additional five percentage points compared to random selection. However, this result should not discourage us as we are more interested in the binary classification case where we want to distinguish between two cases of infected samples. The information that samples are infected is available by examining the samples, as tissue necrosis can be clearly distinguished from healthy regions.

In Table 3, we present the logistic regression classification model performance results for distinguishing between two considered sample types of acorns infected with *Phytophthora* and *Pythium*. As we have noted in previous studies [7], the models built on the data collected from a single sensor can perform better than the models in which the data from all sensors are used. This observation is confirmed in the present studies. The best results can be obtained when the modelling features are extracted from the responses of the TGS 2603 sensor, which is designed for odour and air contaminant detection with applications in air purifiers, ventilation control, deodorization control and air quality monitors. Such a sensor is expected to respond to metabolites emitted from oomycetes or secondary metabolites emitted from infected acorns. The same or similar molecules are responsible for unpleasant odours and should be detected.

It may be interesting to see what modelling features were selected as the predictors for the best-performing classification models. As we already mentioned, we compared the results of modelling when data extracted from all sensors are available for model building with the case when only data from one sensor were used. We also performed several tests using subsets of features allowing selection, for example, only features from the adsorption phase versus features from the adsorption and desorption phase. Moreover, when features were selected from both conductance and resistance curves versus cases of features extraction from only conductance or only resistance, were included. As we have described above, we applied the CV procedure and created multiple classification models. Thus, for each of them, different features were selected. However, we observed that they are similar and the most frequently were the features describing the shape of the derivative calculated from the adsorption part of the response curve. That was the extreme value of the derivative and time to reach this extreme skewness, kurtosis and standard deviation. Moreover, the value reached at the end of the adsorption phase was often selected by the models.

### 3.4. Discussion

In recent years, the role of volatile organic compounds as a cost-effective pest control method has gained importance, reducing the use of chemicals. The search for a cheap and practical device, which we hope is the e-nose, is part of this trend and will be a helpful tool used in Integrated Plant Management’s strategy embodied in the directive European Commission. According to this idea, all physical and biological methods should take precedence over chemical methods. VOCs from microorganisms are also chemical compounds, but the risk of contaminating the soil by them like inorganic chemical pesticides is very low. Therefore, they can be used primarily in nurseries to induce genes responsible for plant defence and mechanisms to prevent infection/disease by pathogens [47]. Moreover, synergism of many VOCs has been found to increase the spectrum of action on pathogens and VOCs promote plant growth [48,49,50]. Studies by Schulz-Bohm et al. [51] and Tilocca et al. [52] identified many VOCs that exhibit antimicrobial activity and increase plant biomass.

The chemical constituents that form volatile antifungal molecules include alcohols, aldehydes, ketones, alkanes, alkenes, amines and benzene, all of which were found in our analysis. The compound 2-ethylhexanal (C${}_{8}$H${}_{16}$O), octan-2-one and octan-3-one are among the ketones found in volatile organic compounds [53] and have the potential for use against microorganisms and fungi [54]. While fatty acids are less effective than the listed compounds and chemical fungicides, their antimicrobial activity has also been reported [55]. Volatile organic compounds have been shown to induce responses against infections and systemic immunity [34,36,56,57]. Compounds such as 6-amyl-a-pyrrole, 1-octen-3-ol, methyl methylbenzoate and m-cresol induce systemic tolerance to pathogens by disrupting salicylic and jasmonic acid signalling pathways [58,59]. Consequently, there is limited information on plant genes that act on VOCs released by pathogens [58,59]. Previous studies suggested that volatilization of limonene, 3-methylbutanal and undecane significantly affects plant diameter and chlorophyll content [16], as do 3-methylbutan-1-ol, 2-methylbutanol, limonene, camphor, β-cedrene and α-bergamotene, which are designated natural volatile compounds [60]. Low concentrations of 2-ethylhexanal promote Arabidopsis growth, whereas high concentrations impair plant growth [61,62,63].

Using the GC-MS method, we could not identify all the chemical compounds listed above known for their activity against microorganisms, but this was not the aim of this work. We mainly wanted to find those that are characteristic of infestation by the two pathogens studied in order to be able to detect them in the samples tested in vitro and then in the nurseries in vivo. The latter will be the subject of our further research. In this article, we also try to draw attention to the practical aspect of our research. The most important practical distinction between organisms of the genus *Pythium* and *Phytophthora* (which we have succeeded in making) is that the nursery forester does not need to take any action when there is an infestation of *Pythium* because there is little risk of the seedlings dying. The situation is quite different when species of the genus *Phytophthora* are found, in which case the action must be taken immediately because there is a risk of significant economic loss. There are always many *Pythium* species in the soil and the seeds come into contact with them, but if the germinating seeds are “sick” because they are infected with introduced *Phytophthora* species (in Latin “plant killers”), the situation requires immediate protection of the seedlings with chemicals. For now, we are pleased to report that we can distinguish between the two oomycetes *Pythium* and *Phytophthora* in vitro based on the signals detected by the gas sensors. An encouraging result for further research is the ability shown in Figure 5 to distinguish samples of healthy acorns from acorns infected with the pathogen *Phytophthora plurivora*. We also believe that an important result of our experimental work is the finding that it is more challenging to detect samples infected with *Pythium* compared to *Phytophthora*. Healthy and “diseased” acorns inoculated with *Pythium* are likely to excrete similar volatile compounds, supporting the thesis that *Pythium* is not a problem because germinating acorns are not infected and consequently do not die. It would be a significant achievement to develop a ready-made tool used by nursery foresters or other plant protection services. The results presented in this publication confirm that we are on the right track and we would like to share them with the scientific community and forest managers.

## 4. Summary and Conclusions

Research in vitro has revealed possibilities for identifying pathogenic oomycetes in infected plant material thanks to an electronic nose, thus taking a significant step towards becoming a valuable tool to assist nurseries and quarantine services. Previously, species of *Phytophthora plurivora*—one of the most common pathogens in nurseries—and *Pythium intermedium* (less pathogenic) were successfully distinguished in germinated acorns of English oak *Quercus robur* L., an important forest tree species in Poland. The analyses of chemical compounds performed by HS-SPME/GC-MS revealed volatile molecules produced by oak seedlings, mainly belonging to terpenes and alkanes. Compounds characteristic only of *Pythium* or *Phytophthora* were also found. Methylcarveol occurred when germinated acorns were infected with *Pythium*, while neophytadiene (isomer 2 and 3) occurred only when infected with *Phytophthora*. Moreover, isopentanol was found in acorns infected with *Phytophthora*, while in the control isopentyl vinyl ether was not observed anywhere else.

We want to emphasize that this is the next stage of work (after testing in vitro on pure cultures of pathogens) and these results look encouraging, although the actual application is still a long way off. We are currently improving the device and testing it under in vivo conditions on healthy and infected germinating acorns in jars. Oaks bear fruit every few years, so it is necessary to store them for continuity of forest restoration work. During the winter, acorns harvested and stacked on the wood floor (in sheds) are infested with pathogens despite periodic shuffling. Sampling acorns on this occasion and evaluating them for the presence of oomycetes allows their early detection, saving nurseries from losses. Similarly, acorns sown in spring (or autumn) can be tested for particularly dangerous pathogens of the genus *Phytophthora*. From this point of view, it is essential to distinguish between a strong pathogen such as *Phytophthora plurivora* and a weak one such as *Pythium intermedium*. This helps the grower to decide whether to use fungicides or not.

The desired goal of the undergoing research will be in vivo testing of e-nose in forest nurseries. The sustainability and diversity of future forests depend on the quality of propagation material, so it should be free of harmful organisms. It is the final stage where unwanted alien (invasive) organisms can be eliminated. Once they have invaded plantations and stands, eradication is too costly, if feasible at all. E-noses help select healthy nursery stock free of pathogens, especially when plants are symptomless due to pesticide use. When planted under favourable environmental conditions (riparian or floodplain), dormant pathogens begin their activity and cause disease.

There is an essential difference between the GC-MS and electronic nose approaches that should be noted. The first one is an analytical technique that allows identifying the individual chemical components of the measured sample. It also allows measuring the concentration of these components. On the other hand, the electronic nose and machine learning software are based on pattern recognition of the measured signals and do not provide any information about the chemical composition. It uses a training set of observations to learn patterns which can be later used to discriminate between the categories using measurements of new measurement data. These data can also be used to indicate deviations from already recognised patterns.

## Figures and Tables

**Table 1:** The confusion matrix elements used to define metrix of classification models performance.

		Actual	
		Positive	Negative
Predicted	Positive	tp (true positive)	fp (false positive)
	Negative	fn (false negative)	tn (true negative)

**Table 2:** The VOCs differentiating the measured samples of acorns infected by the *Phytophthora* and *Pythium* oomycetes, detected by the Gas Chromatography-Mass Spectrometry method. Meaning of the table columns is provided in Appendix A Table A2.

Compound	CAS	m/z	M${}^{+}$	$t_{\mathrm{ret}.}$ (min)	${\mathrm{RI}}_{\exp.}$	${\mathrm{RI}}_{\mathrm{lit}.}$	TIC (%)
***Phytophthora***							
Neophytadiene isomer 2	-	68, 82, 95, 43, 57	278	31.723	1864	1864	0.28
Neophytadiene isomer 3	-	68, 82, 95, 43, 57	278	32.087	1882	1882	0.53
Isopentanol	123-51-3	55, 41, 42, 70, 43	88	3.534	723	726	0.65
***Pythium***							
Methylcarveol	85710-64-1	43, 41, 109, 83, 55	166	12.683	1091	n/a	1.43

**Table 3:** Performance of classification models with logistic regression trained with features extracted from responses of all sensors and only the single sensor TGS 2603. Cross-validation in groups determined by the day of measurements. Binary target classification of sample categories of acorns infected with *Phytophthora* and *Pythium*.

	All Sensors	One Sensor
accuracy	58%	64%
precision of *Phytophthora*	56%	60%
precision of *Pythium*	59%	64%
recall of *Phytophthora*	60%	64%
recall of *Pythium*	55%	68%

## Data Availability

The data presented in this study are available from the corresponding author.

## Appendix A. Results of the Gas Chromatography-Mass Spectrometry Measurements

**Table A1:** The chemical components identified by the Gas Chromatography-Mass Spectrometry measurements. By the blue color are highlighted the components specific to the considered category of samples. The meaning of columns is defined in Table A2.

							Healthy		Phytophthora		Pythium	
Compound	CAS	m/z	$M^{+}$	t${}_{\mathrm{ret}.}$ (min)	${\mathrm{RI}}_{\exp.}$	${\mathrm{RI}}_{\mathrm{lit}.}$	Area $\times 10^{6}$	TIC (%)	Area $\times 10^{6}$	TIC (%)	Area $\times 10^{6}$	TIC (%)
**Alkanes**							601.6	71.67	608.3	68.12	623.4	71.63
including:												
n-Butane	106-97-8	43, 41, 58, 42, 44	58	1.706	400	400 ${}^{a}$	20.7	2.47	30.2	3.39	34.8	4.00
2.3.5-Trimethylhexane	1069-53-0	43, 41, 85, 84, 57	128	5.003	808	810 ${}^{b}$	2.4	0.29	2.5	0.29	2.2	0.26
2.4-Dimethylheptane	2213-23-2	43, 41, 85, 57, 71	128	5.163	815	818 ${}^{b}$	59.0	7.03	34.3	3.84	52.9	6.09
4-Methyloctane	2216-34-4	43, 41, 85, 71, 84	128	6.163	858	n/a	5.3	0.64	5.5	0.62	4.1	0.48
n-Decane	124-18-5	57, 43, 71, 85, 41	142	9.984	1000	1000 ${}^{a}$	21.4	2.55	13.2	1.48	22.7	2.61
2.6-Dimethylnonane	17302-28-2	43, 57, 71, 41, 85	156	10.425	1019	1022 ${}^{b}$	10.7	1.28	6.8	0.77	7.8	0.90
5-Methyldecane	13151-35-4	43, 57, 71, 41, 85	156	11.156	1054	1057 ${}^{b}$	15.6	1.87	15.2	1.71	11.0	1.27
4-Methyldecane	2847-72-5	41, 71, 57, 41, 70	156	11.431	1056	1059 ${}^{b}$	15.6	1.87	15.8	1.78	5.2	0.61
Alkane C${}_{12}$H${}_{26}$	-	43, 57, 71, 41, 85…155, **170**	170	11.671	1057	-	89.4	10.66	87.5	9.81	74.4	8.55
Alkane C${}_{12}$H${}_{26}$	-	43, 57, 71, 41, 85…155, **170**	170	11.829	1063	-	18.6	2.22	20.8	2.33	15.4	1.78
n-Undecane	1120-21-4	57, 43, 71, 85, 41	156	12.990	1100	1100 ${}^{a}$	28.1	3.35	28.4	3.19	30.6	3.52
n-Dodecane	112-40-3	57, 43, 71, 85, 41	170	15.969	1200	1200 ${}^{a}$	15.4	1.84	16.3	1.83	14.5	1.68
Alkane C${}_{14}$H${}_{30}$	-	43, 57, 71, 41, 85, …, 183, **198**	198	16.207	1214	-	15.8	1.89	19.0	2.13	17.4	2.01
Alkane C${}_{14}$H${}_{30}$	-	43, 57, 71, 41, 85, …, 183, **198**	198	17.065	1245	-	15.3	1.83	18.2	2.04	17.8	2.05
Alkane C${}_{14}$H${}_{30}$	-	43, 57, 71, 41, 85, …, 183, **198**	198	17.214	1250	-	16.2	1.93	18.2	2.04	17.1	1.98
Alkane C${}_{14}$H${}_{30}$	-	43, 57, 71, 41, 85, …, 183, **198**	198	17.369	1256	-	23.4	2.79	20.8	2.34	28.8	3.32
Alkane C${}_{14}$H${}_{30}$	-	43, 57, 71, 41, 85, …, 183, **198**	198	17.611	1265	-	26.1	3.12	31.4	3.52	37.6	4.32
2.6.11-Trimethyldodecane	31295-56-4	43, 57, 71, 41, 85	212	17.890	1275	1275 ${}^{b}$	9.3	1.11	10.9	1.22	6.3	0.73
Alkane C${}_{15}$H${}_{32}$	-	43, 57, 71, 41, 85, …, 197, **212**	212	18.287	1289	-	18.6	2.22	22.5	2.52	20.9	2.41
Alkane C${}_{15}$H${}_{32}$	-	43, 57, 71, 41, 85, …, 197, **212**	212	18.439	1295	-	22.9	2.74	24.3	2.73	25.7	2.95
n-Tridecane	629-50-5	57, 43, 71, 85, 41	186	18.645	1300	1300 ${}^{a}$	13.9	1.66	16.8	1.88	15.8	1.82
Alkane C${}_{15}$H${}_{32}$	-	43, 57, 71, 41, 85, …, 197, **212**	212	19.293	1327	-	36.6	4.36	35.5	3.98	46.0	5.29
n-Tetradecane	629-59-4	57, 43, 71, 85, 41	198	21.201	1400	1400 ${}^{a}$	3.7	0.45	3.2	0.37	4.4	0.51
Alkane C${}_{16}$H${}_{34}$	-	43, 57, 71, 41, 85, …, 211, **226**	226	21.778	1423	-	12.4	1.49	12.8	1.43	10.4	1.20
Alkane C${}_{16}$H${}_{34}$	-	43, 57, 71, 41, 85, …, 211, **226**	226	22.761	1462	-	7.0	0.85	9.3	1.04	9.8	1.13
Alkane C${}_{16}$H${}_{34}$	-	43, 57, 71, 41, 85, …, 211, **226**	226	22.920	1469	-	7.5	0.90	7.6	0.86	7.5	0.87
Alkane C${}_{16}$H${}_{34}$	-	43, 57, 71, 41, 85, …, 211, **226**	226	23.462	1491	-	7.5	0.90	10.9	1.23	10.7	1.24
n-Pentadecane	629-62-9	57, 43, 71, 85, 41	212	23.694	1500	1500 ${}^{a}$	42.8	5.11	52.8	5.91	45.5	5.23
Alkane C${}_{17}$H${}_{36}$	-	43, 57, 71, 41, 85, …, 225, **240**	240	24.681	1542	-	12.4	1.49	12.8	1.44	16.6	1.91
Alkane C${}_{19}$H${}_{40}$	-	43, 57, 71, 41, 85, …, 253, **268**	268	28.515	1711	-	6.5	0.78	3.5	0.40	8.0	0.93
**Terpenes**							**169.2**	**20.16**	**219.7**	**24.60**	**177.8**	**20.43**
including:												
1,2-Dimethyl-5-prop-1-en-2-ylcyclohex-2-en-1-ol (methylcarveol)	85710-64-1	43, 41, 109, 83, 55	166	12.683	1091	n/a	-	-	-	-	**12.4**	**1.43**
2,6,10-Trimethyldodecane (farnesane)	3891-98-3	43, 57, 71, 41, 85	212	18.068	1281	1282 ${}^{a}$	121.6	14.49	144.2	16.15	125.3	14.40
6,8α-Dimethyl-3-propan-2-yl-2,4,5,8-tetrahydro-1H-azulene (daucene)	16661-00-0	161, 121, 162, 91, 93	204	20.774	1383	1380 ${}^{a}$	5.2	0.63	24.6	2.76	6.2	0.72
(1S,8αR)-4,7-Dimethyl-1-propan-2-yl-1,2,3,5,6,8α-hexahydronaphthalene (δ-cadinene)	483-76-1	161, 204, 119, 105, 134	204	24.251	1524	1522 ${}^{c}$	4.3	0.51	2.4	0.28	2.3	0.27
(3αS,8αS)-6,8α-Dimethyl-3-propan-2-ylidene-1,2,3α,4,5,8-hexahydroazulene (dauca-4(11),8-diene)	395070-76-5	136, 121, 41, 204, 91	204	24.500	1532	1530 ${}^{c}$	6.9	0.82	7.4	0.83	4.2	0.49
Sesquiterpenoid C15H26O	-	59, 149, 107, 91, 93…**222**	222	25.924	1594	-	9.9	1.18	6.2	0.70	5.4	0.63
2-[(2R,4αR,8αS)-4α-Methyl-8-methylidene-1,2,3,4,5,6,7,8α-octahydronaphthalen-2-yl]propan-2-ol (β-eudesmol)	473-15-4	59, 149, 41, 109, 43	222	27.324	1651	1649 ${}^{c}$	4.9	0.59	3.8	0.44	3.2	0.38
2-[(2R,4αR,8αR)-4α,8-Dimethyl-2,3,4,5,6,8α-hexahydro-1H-naphthalen-2-yl]propan-2-ol (α-eudesmol)	473-16-5	59, 149, 161, 204, 189	222	27.388	1654	1652 ${}^{c}$	12.6	1.51	15.3	1.72	14.3	1.65
7,11,15-Trimethyl-3-methylidenehexadec-1-ene (neophytadiene), isomer	504-96-1	68, 82, 95, 43, 57	278	31.219	1839	1840 ${}^{d}$	3.5	0.42	8.2	0.93	4.0	0.47
Neophytadiene, isomer	-	68, 82, 95, 43, 57	278	31.723	1864	1864 ${}^{d}$	-	-	**2.5**	**0.28**	-	-
Neophytadiene, isomer	-	68, 82, 95, 43, 57	278	32.087	1882	1882 ${}^{d}$	-	-	**4.7**	**0.53**	-	-
**Other compounds**							**14.1**	**1.68**	**14.6**	**1.64**	**9.0**	**1.04**
including:												
3-Methylbutan-1-ol (isopentanol)	123-51-3	55, 41, 42, 70, 43	88	3.534	723	726 ${}^{b}$	-	-	**5.8**	**0.65**	-	-
1-Ethenoxy-3-methylbutane (isopentyl vinyl ether)	39782-38-2	43, 70, 55, 41, 71	114	4.052	754	n/a	**4.9**	**0.59**	-	-	-	-
2,4-Dimethylhept-1-ene	19549-87-2	43, 70, 55, 41, 39	126	5.620	840	842 ${}^{b}$	4.9	0.59	3.9	0.44	3.7	0.43
2,2-Dimethylbutan-1-ol	1185-33-7	43, 71, 41, 29, 70	102	5.783	842	n/a	4.1	0.49	4.8	0.55	5.2	0.60
**Undefined compounds**							**54.4**	**6.49**	**50.2**	**5.63**	**60.0**	**6.90**
including:												
NN	-	133, 151, 135, 134, 77	-	7.256	904	-	36.9	4.40	22.1	2.48	35.0	4.03
NN	-	43, 69, 111, 55, 75	-	20.291	1365	-	17.5	2.09	28.1	3.15	25.0	2.88

^*a*^—standards. ^*b*^—NIST (2020). ^*c*^—Adams (2007). ^*d*^—Tkachev (2008). n/a—non available

**Table A2:** Description of the columns in tables presenting the Gas Chromatography-Mass Spectrometry results.

Compound	Group and Name of the Identified Compounds
CAS	CAS Registry Number.
m/z	Mass-to-charge ratio (fragmentation ion).
M${}^{+}$	Molecular ion.
t${}_{ret.}$	Retention time.
RI${}_{exp.}$	Experimental value of the Retention Index.
RI${}_{lit.}$	Literature value of the Retention Index.
TIC	Percentage of the Total Ion Current.
